# Optimizing variant-specific therapeutic SARS-CoV-2 decoys using deep-learning-guided molecular dynamics simulations

**DOI:** 10.1038/s41598-023-27636-x

**Published:** 2023-01-14

**Authors:** Katharina Köchl, Tobias Schopper, Vedat Durmaz, Lena Parigger, Amit Singh, Andreas Krassnigg, Marco Cespugli, Wei Wu, Xiaoli Yang, Yanchong Zhang, Welson Wen-Shang Wang, Crystal Selluski, Tiehan Zhao, Xin Zhang, Caihong Bai, Leon Lin, Yuxiang Hu, Zhiwei Xie, Zaihui Zhang, Jun Yan, Kurt Zatloukal, Karl Gruber, Georg Steinkellner, Christian C. Gruber

**Affiliations:** 1 Innophore GmbH, 8010 Graz, Austria; 2 Austrian Centre of Industrial Biotechnology, 8010 Graz, Austria; 3 Institute of Molecular Bioscience, University of Graz, 8010 Graz, Austria; 4 SignalChem Lifesciences Corp., 110-13120 Vanier Place, Richmond, BC V6V 2J2 Canada; 5 Diagnostic- and Research Center for Molecular Biomedicine, Institute of Pathology, Medical University of Graz, 8010 Graz, Austria; 6 Field of Excellence BioHealth, University of Graz, 8010 Graz, Austria

**Keywords:** Viral infection, Recombinant protein therapy, Machine learning, Protein function predictions, Virtual drug screening

## Abstract

Treatment of COVID-19 with a soluble version of ACE2 that binds to SARS-CoV-2 virions before they enter host cells is a promising approach, however it needs to be optimized and adapted to emerging viral variants. The computational workflow presented here consists of molecular dynamics simulations for spike RBD-hACE2 binding affinity assessments of multiple spike RBD/hACE2 variants and a novel convolutional neural network architecture working on pairs of voxelized force-fields for efficient search-space reduction. We identified hACE2-Fc K31W and multi-mutation variants as high-affinity candidates, which we validated in vitro with virus neutralization assays. We evaluated binding affinities of these ACE2 variants with the RBDs of Omicron BA.3, Omicron BA.4/BA.5, and Omicron BA.2.75 in silico. In addition, candidates produced in *Nicotiana benthamiana*, an expression organism for potential large-scale production, showed a 4.6-fold reduction in half-maximal inhibitory concentration (IC_50_) compared with the same variant produced in CHO cells and an almost six-fold IC_50_ reduction compared with wild-type hACE2-Fc.

## Introduction

The coronavirus disease 2019 (COVID-19) pandemic, caused by severe acute respiratory syndrome coronavirus 2 (SARS-CoV-2), is combated twofold: with vaccination programs and with therapeutics, such as the SARS-CoV-2 main protease inhibitor nirmatrelvir/ritonavir (Paxlovid™)^1^. Due to the ongoing evolution towards escape of existing immunity^2,3^, treatment options that are independent of spike protein mutations or that are easily adaptable constitute a significant piece of the puzzle to reduce the virus’s danger, not only for the unvaccinated part of the population^4^. In a similar fashion to the appearance of the Beta (B.1.351), Gamma (P.1), and recently Omicron (B.1.1.529) variants^5–8^, yet other SARS-CoV-2 immune-escape variants may emerge in the future.

While there are several candidate receptors for the initial binding of SARS-CoV-2 to host cell surfaces, the attachment is mainly mediated via binding of its trimeric spike proteins to the cellular receptor angiotensin-converting enzyme 2 (ACE2). Host membrane fusion is enabled by proteolytic processing of the spike by host proteases like furin, TMPRSS2, and cathepsin B/L^9–11^. The interplay between these events enables the virus to enter the host cell and to subsequently replicate^3,12^. Besides the diversity of host proteins binding to SARS-CoV-2 and the efficient proteolytic cleavage of the spike protein, a major reason for the virus’ high transmissibility is suggested to be the strong binding between the spike receptor-binding domain (RBD) and ACE2^11^. Thus, one promising way to prevent infection is to target the entry mechanism by blocking the interaction between the viral spike RBD and human ACE2 (hACE2).

Human soluble ACE2 (hsACE2) has proven to be a therapeutically potent decoy to block SARS-CoV-2 infection in human capillary organoids and COVID-19 model hamsters^13,14^. The application of recombinant hsACE2 has been reported to be safe in healthy human subjects^15^ but did not reduce mortality in a phase 2 trial (ClinicalTrials.gov ID: NCT04335136).

There have been various approaches to modify the hsACE2 protein to increase its moderate binding affinity to the spike RBD (K_D_ ≈ 20 nM)^16–19^. Using the hACE2 dimer (hACE2_2_) instead of a monomer enhanced its effect against SARS-CoV-2 infection due to avidity effects^18,20^. Fusion to segments of human IgG improved its pharmacokinetics in mouse models^21^ and activated degranulation of NK cells^22^. In addition, mutations at hotspot positions e.g. S19, T27, K31, H34, L79, and N330 enhanced hACE2’s affinity for the SARS-CoV-2 spike RBD^18,20,23–25^. Promising starting positions for hACE2 affinity optimizations have been identified through extensive experimental screening procedures such as deep mutagenesis^18^ or a computational approach based on the local shape characterization of protein surfaces^26^. Treatment with a hsACE2_2_ triple mutant reduced mortality in SARS-CoV-2 infected K18-hACE2 mice^27^. These various hACE2 modifications improved the binding affinity to a KD in the low nanomolar to subnanomolar range^20,28^. In the future, virus-binding ACE2 elements from high-affinity candidates could be assembled to form small peptide inhibitors, applied as inhaled therapeutics against COVID-19, benefiting from their low molecular weights^29^.

Based on these observations and data, we devised a workflow using a combination of standard techniques and our point-cloud technology: First, we used extensive molecular dynamics (MD) simulations to identify hACE2 mutations that strengthen the interaction with the spike RBD. To this end, we employed an empirical scoring function (ESF) closely related to the linear interaction energy (LIE) method, which has been calibrated on experimental binding data, as described previously^30^. We performed virus-neutralization assays to evaluate the potential of four hACE2 variants linked to an Fc segment of human IgG1 (hACE2-Fc) to inhibit the spread of wild-type SARS-CoV-2 as well as SARS-CoV-2 Beta, which has acquired mutations that reduced the binding to class 1 antibodies^5^. In addition, we also expressed some of the hACE2-Fc variants in *Nicotiana benthamiana* to test whether mass production would be feasible. Properties such as a fast growth rate and a natural ability to express heterologous gene sequences make *N. benthamiana* particularly well suited to the production of therapeutic proteins^31^.

The MD-simulation data were combined with hACE2- and spike RBD Cataphore Halos^30^ to train an artificial neural network (ANN). Our deep-learning models presented here are intended as a tool to predict binding affinities of the SARS-CoV-2 spike protein with hACE2 variants. Apart from that, the models should be able to predict the hACE2-binding affinities for spike RBDs of newly emerging SARS-CoV-2 variants based solely on hACE2 and spike RBD Halos. When new virus mutants emerge, the vast mutational landscape of hACE2 could then rapidly be screened via the ANN, and the top performers verified using MD so that the treatment could be adapted to the hsACE2 variant with the highest binding affinity to the presently dominant SARS-CoV-2 variant, or even optimized to several important variants.

## Results

### Overall strategy

Deep-mutational scans examining a broad range of spike RBD and hACE2 single mutants have shown severe alterations in binding and expression properties^18,32^. We collected high-affinity hACE2 mutants from literature and combined them with mutations identified by visual examination of the hACE2-RBD binding interface.

We then optimized an ESF, as described previously^30^, to screen the collection for hACE2 mutants with enhanced binding affinities to the spike RBD from SARS-CoV-2 variants of concern (VOCs). Furthermore, we trained an ANN on our MD simulation data to produce binding affinity estimates for many variants of both spike RBD and hACE2 much faster than our approach based on the ESF. Thus this ANN will mainly serve as a future high-throughput filter for new spike RBD variants.

**Figure 1:**
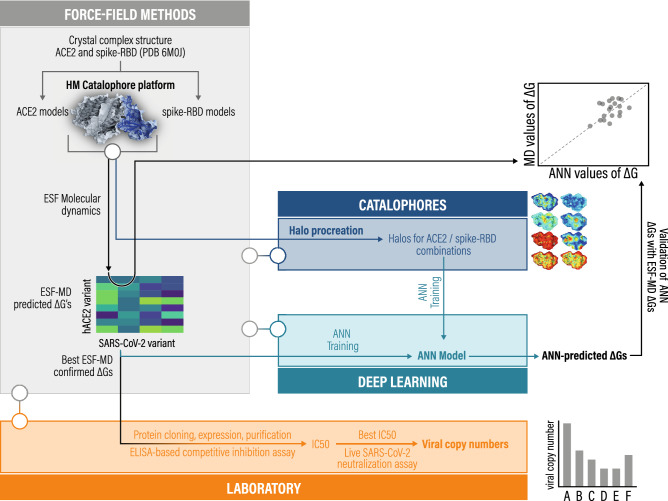
Multi-level strategy to identify high-affinity human ACE2 (hACE2) variants for neutralization of diverse SARS-CoV-2 variants. In brief, homology models (HM) of varying SARS-CoV-2 spike receptor binding domain (RBD) structures in complex with hACE2 variant structures served as input for molecular dynamics (MD) simulations analyzed via an empirical scoring function (ESF) closely related to the linear interaction energy (LIE) model. Gibbs free energy predictions (ΔG) were combined with Catalophore Halos to train an artificial neural network (ANN). This enables the model to predict ΔG values for hACE2- and SARS-CoV-2 variants based on their Halos. ANN ΔG predictions were validated with ESF ΔG values. The SARS-CoV-2 neutralizing potential of promising hACE2 variants was evaluated in ELISA-based competitive inhibition assays followed by live SARS-CoV-2-based neutralization assays.

Promising hACE2 variants from our simulations were then expressed in CHO cells and *N. benthamiana* plant leaves. Their SARS-CoV-2-neutralizing capacity was evaluated in wet-lab experiments. Figure 1 provides a visualization of our integrated computational and experimental strategy.

### ESF binding energy predictions correlate with experimental data

A LIE model is an efficient way to compute binding energy differences from ensembles of short MD simulations of different ligands in a binding site. The LIE model can also be used to calculate the Gibbs free energy difference between a free ligand in water and the ligand in complex with a protein. In a previous publication^30^, we applied an ESF derived from the LIE model to the SARS-CoV-2 spike RBD as the “protein” together with the hACE2 as the “ligand”. Although this seems somewhat arbitrary at first glance, we tested hACE2 and spike RBD as ligands and found the former to be more reliable in terms of model stability. In this paper, we will apply the same approach to a different dataset.

In particular, we validated our existing ESF using a subset of hACE2 variants by examining the correlation between predicted Gibbs free energy values (Δ*G*_pred_) and experimental half-maximal effective concentration (EC_50_) values deriving from in vitro binding affinity experiments (Table S1, Fig. S1). We found that the experimental data were well-correlated with predicted Gibbs free energies from the model (*R*^2^ = 0.54).

### ESF reveals high-affinity hACE2 variants

**Figure 2:**
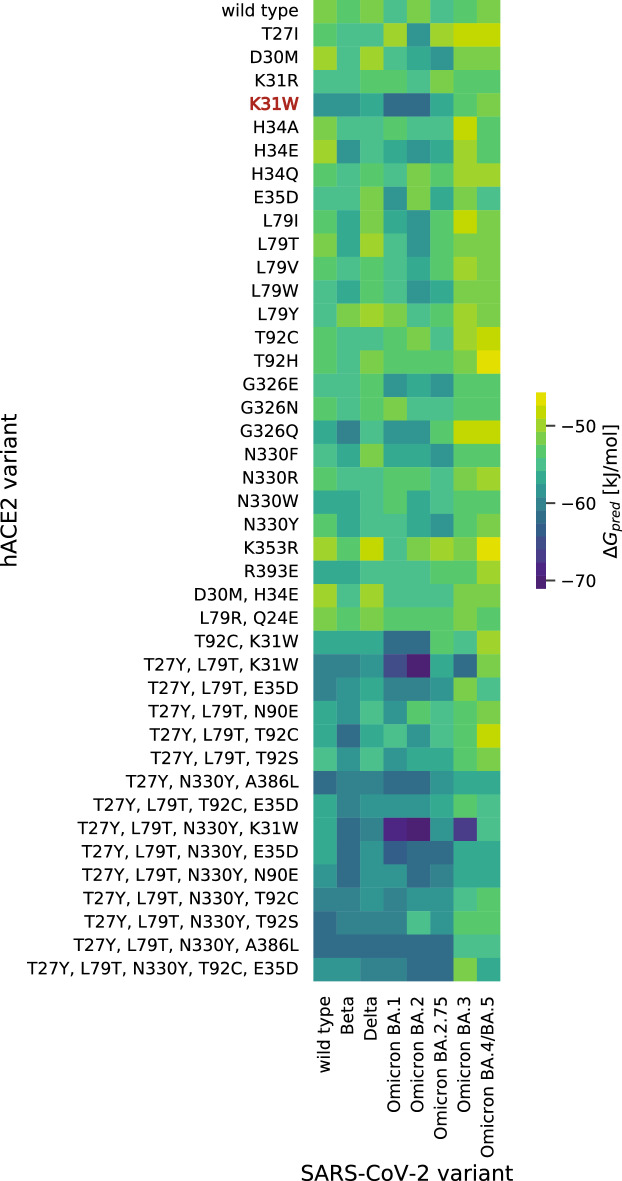
Gibbs free energy predictions of preselected hACE2 variants in combination with SARS-CoV-2 variants of concern. Van der Waals (vdw) and electrostatic (elec) energies from the MD simulation in combination with optimal fitted weights (*ω*^elec^ = 0.024, *ω*^vdw^ = 0.765) from the training set resulted in predicted Gibbs free energies (Δ*G*_pred_).

Compared to wild-type hACE2, most preselected hACE2 variants show enhanced binding affinities to the five SARS-CoV-2 variants: wild type, Beta, Delta, Omicron BA.1, and Omicron BA.2 (Figs. 2, S2). Since hACE2-K31W is the only single mutant with strikingly low Δ*G*_pred_ values, K31W might play a key role in the interaction with the spike RBD. The K31W mutation is also present in the top high-affinity multi-mutants. Mutants with three to five amino acid substitutions showed the highest binding affinities to the spike RBD. Reaching Δ*G*_pred_ values around − 71 kJ/mol, hACE2 T27Y_L79T_N330Y_K31W and hACE2 T27Y_L79T_K31W reveal exceptionally high affinities to the Omicron BA.2 spike RBD compared to − 52 kJ/mol for the wild-type hACE2. In addition, we included the recently emerged Omicron sublineages BA.3, BA.4/BA.5, and BA.2.75 in our hACE2-RBD binding affinity evaluation. With predicted binding affinities of − 67 kJ/mol and − 62 kJ/mol, hACE2 T27Y_L79T_N330Y_K31W and hACE2 T27Y_L79T_K31W remained the top high-affinity variant for Omicron BA.3, whereas the effect diminished for BA.4/BA.5 and BA.2.75 (Figs. 2, S2).

### Halo procreation and deep-learning data preparation

**Figure 3:**
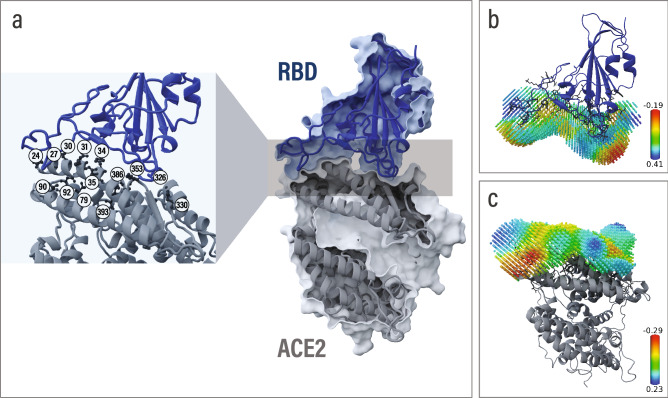
The binding interface between SARS-CoV-2 spike RBD, shown in dark blue, and hACE2, visualized in gray (PDB: 6M0J). (**a**) Selection of hACE2 mutations from MD simulations and binding affinity assays are shown as sticks. Mutated amino acids are labeled according to their position in the hACE2 protein chain. (**b**) SARS-CoV-2 spike RBD and spike RBD Hydrophobicity Halo. (**c**) hACE2 and hACE2 Hydrophobicity Halo.

Catalophore Halos are fields of physicochemical properties defined on a 3D point cloud above the protein’s surface and are computed from a protein’s structure^30^. For our experiments, we procreated Halos for every hACE2 and spike RBD variant at the spike RBD/hACE2 binding interface. The deep-learning models use the Halo of a spike RBD and the Halo of a hACE2 (visualized in Figs. 3, S3) to predict the binding affinity of these two proteins. The fact that the ANN is trained on MD-simulation data leads to a reliable deep-learning setup since both the MD-simulation results and the ANN predictions rely on input data from the same source. The MD simulations use the structural information of the protein, and the ANN uses the Halos, which are also derived directly from the protein structures. After their computation, Halos are considered independent from the amino-acid sequence and represent the functional capacities of the protein’s direct surrounding area.

### ANN predictions and validation

ANN predictions were implemented in two different ways. Firstly, the system’s potential to predict the effects of small mutational changes in spike RBD variants for the same hACE2 decoys was investigated using Omicron BA.1 and BA.2 as examples. Secondly, a large-scale screening of the mutational landscape spanned by all possible single-point mutations of hACE2 was performed in combination with the Omicron BA.2 spike RBD variant.

As a first step, we trained our ANN on different data sets in order to explore its capabilities in predicting variants with unseen combinations of amino-acid exchanges, both on the spike RBD and the hACE2 side of the binding complex. Details of these experiments are given in the Methods section below.

**Figure 4:**
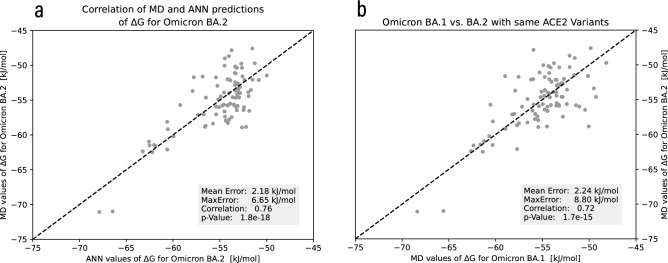
Basic comparison to gauge the potential of the ANN prediction model. (**a**) Correlation plot between Gibbs free-energy values (Δ*G*) from MD simulation and ANN model prediction for SARS-CoV-2 Omicron BA.2 on a set of hACE2 variants (Pearson correlation = 0.76). (**b**) Correlation of Omicron BA.1 and BA.2 Δ*G* values on the same hACE2 receptors (Pearson correlation = 0.72).

To test the predictive power of such a network and setup, we used the ANN to predict binding affinities of the Omicron BA.2 spike RBD with several hACE2 variants. Therefore the training was based on the entire ESF dataset except for Omicron-BA.2-variant data. In order to gauge the potential of an ANN prediction model, we made the following observations:

The pure Pearson-correlation between MD results for Omicron BA.1 and BA.2 variants, respectively, given the same set of hACE2 receptors, is 0.72. Remarkably, the correlation between MD simulated BA.2 and the model-predicted BA.2 increases to 0.76, indicating that the model explains an additional 6.9% of the variance compared to a pure extrapolation from BA.1 variants. When removing the two most potent binding variants with values < − 70 kJ/mol, the correlations change to 0.60 and 0.66, respectively, while the difference in explained variance increases to 7.5%. The model also exhibited lower worst-case performance, as the maximum error for the pure correlation between BA.1 and BA.2 was 8.80 kJ/mol versus the one obtained by the model at 6.65 kJ/mol. Most of the worst performers are in the lower end of > − 50 kJ/mol, whereas the predictive power for high binding affinities is of much more interest for the present paper and our ambition in general. Furthermore, the model mapped the highest outliers, whose MD Δ*G* values were < − 70 kJ/mol, to the highest affinity value seen during training (− 68 kJ/mol), and successfully understood that the outlier with the second-highest affinity would also bind stronger to BA.2 spike RBD, as seen in Fig. 4. This prediction shows that the ANN is not only able to better predict the values close to the bulk of the affinity distribution than extrapolating from very closely related variants but also that it reliably maps the strongly underrepresented high-affinity samples to the highest affinity bracket around − 68 kJ/mol.

These observations show that the ANN can learn meaningful physical insights from Halos with a performance significantly better than simply learning a copy-function or regression-to-the-mean for the closest previously seen data. This is especially true for the case of strong binding. Furthermore, since all of the data in the training set were either Omicron BA.1, which is relatively distant from the wild-type spike RBD, or were much closer to the wild type, this indicates that the model is able to combine learned insights from relatively different inputs, in this case rather distant sequences. This is likely an advantage of the sequence- and structure-agnostic nature of the ANN’s Halo training data.

After verifying the predictive power of the ANN, the mutational landscape of hACE2 was screened by predicting all possible single amino-acid exchanges. The 300 most promising predictions were verified using the MD-model. As shown in Fig. S4, the model identified single mutants comparable to the best hACE2 mutant found in the initial MD runs. (For a detailed description, see the “ANN guided ACE2 pre-selection” section in the supplementary information).

### Virus-neutralization experiments are in line with ESF predictions

Before describing our experimental results, it is important to note that a comparison between our ANN- and MD-model predictions and the neutralization experiments is not straightforward. While in silico*,* we studied effects on interaction energies in a well-defined model environment, in vitro results may be influenced by a variety of other factors. Nevertheless, our numerical approach is able to predict binding affinities without using extensive MD calculations, and binding affinity certainly is an important factor of neutralization efficacy.

In addition to wild-type hACE2, we expressed promising hACE2 (residues 19–740) variants from the MD screening with a *C*-terminal human IgG Fc tag in CHO cells. To exclude unintended physiological interactions in the human body^20^, we eliminated hACE2’s peptidase activity by mutating the following positions: H374N and H378N^33^. We conducted BSL-3 virus-neutralization assays to evaluate their potential to block SARS-CoV-2 infections with either wild-type SARS-CoV-2 or SARS-CoV-2 Beta. Remdesivir, an inhibitor of the viral RNA-dependent RNA polymerase^34^, served as a positive-control compound during the assay. Two independent methods, quantification of the SARS-CoV-2 RNA levels in the cell-culture supernatant by quantitative Reverse Transcription PCR (qRT-PCR) and immunohistochemical staining of infected cells using a monoclonal anti-SARS-CoV-2 Nucleocapsid antibody in combination with an HRP-conjugated secondary antibody, were implemented for the analysis of the neutralization experiments. A calibration curve allowed the determination of viral copies in the supernatant based on cycle threshold (Ct) values from the qRT-PCR.

**Figure 5:**
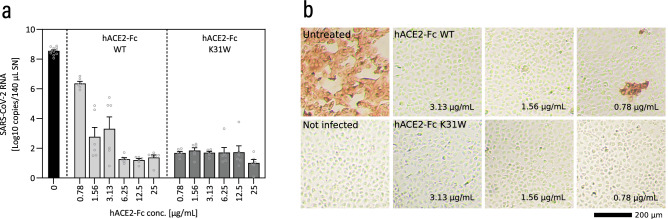
hACE2-Fc K31W inhibits infection by the SARS-CoV-2 Beta variant (MOI: 0.0003) to a greater extent than hACE2-Fc wild type (WT). (**a**), qRT-PCR analysis was used to determine the inhibiting effect of hACE2-Fc WT and hACE2-Fc K31W in concentrations between 0.78 and 25 µg/mL (25–6.25 µg/mL and 3.13–0.78 µg/mL in separate experiments) in virus neutralization assays in VeroE6 cells. No substance (“Untreated”) and no virus (“Not infected”) served as positive and negative controls. Data are presented as mean ± SEM of 6 replicates each. (**b**) Immunohistochemical staining using a monoclonal anti-SARS-CoV-2 Nucleocapsid antibody in combination with a HRP-conjugated secondary antibody was implemented to stain virus-infected cells after the virus neutralization assays with hACE2-Fc concentrations between 3.13 and 0.78 µg/mL. Representative bright-field-microscope images (40 × magnification) of VeroE6 cells are presented.

Preincubation of wild-type SARS-CoV-2 with 25 µg/mL wild-type hACE2-Fc reduced the viral-copy number from 4.95E+08 and 1.66E+08 for the untreated control to 9.51E+04 and 1.03E+03 viral copies for the treated samples, respectively (Fig. S7). hACE2-Fc variants E35D, K31W, and T92C even showed enhanced potential against SARS-CoV-2 infections and led to nearly complete inhibition of infections. A similar effect was obtained for infections with the SARS-CoV-2 Beta variant (Fig. S7). At a final concentration of 0.78 µg/mL (Fig. 5a), a 53,480-fold decrease of viral copies was measured for hACE2-Fc K31W treated samples (5.59E+01 viral copies) compared to hACE2-Fc wild type treated samples (2.99E+06 viral copies). The same effect was also confirmed via immunohistochemical staining of SARS-CoV-2 infected cells (Fig. 5b).

### hACE2-Fc expression in *N. benthamiana* plant leaves

**Figure 6:**
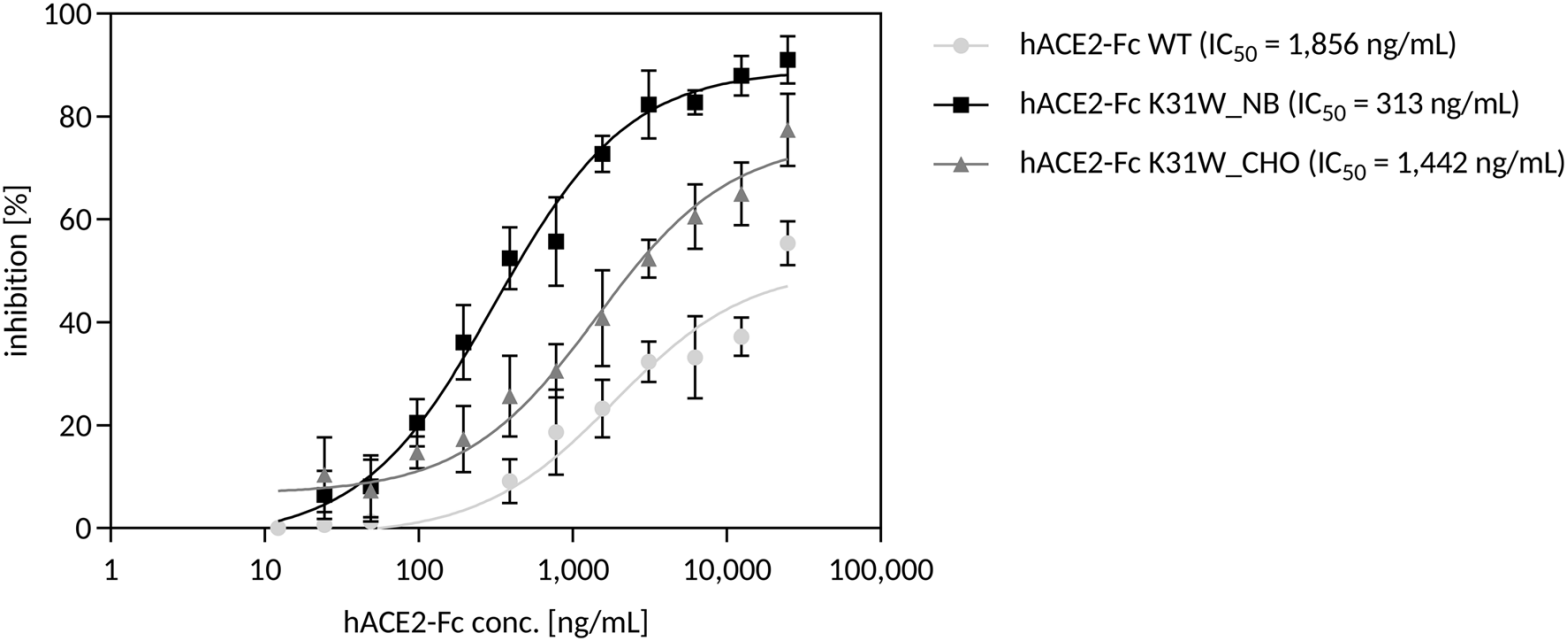
hACE2-Fc K31W expressed in *N. benthamiana* (hACE2-Fc K31W_NB) has higher inhibitory activity against spike RBD binding to immobilized wild-type hACE2-Fc compared to the same variant expressed in CHO cells (hACE2-Fc K31W_CHO) and compared to wild-type hACE2-Fc (hACE2-Fc WT) produced in CHO cells. Soluble hACE2 variants were preincubated with spike RBD protein before being added to the wells of an assay plate precoated with hACE2. The binding of spike RBD to well-surface hACE2 was determined by an ELISA-based competitive inhibition assay in two independent experiments. Data are presented as mean ± SD of duplicates from one representative experiment.

In addition to protein expression in CHO cells, we used *N. benthamiana* plant leaves for hACE2-Fc K31W production and evaluated their spike RBD neutralization potential in ELISA-based competitive inhibition assays. We found that hACE2-Fc K31W produced in *N. benthamiana* leaves has a higher inhibitory activity against spike RBD than hACE2-Fc K31W produced in CHO cells (Fig. 6). Both hACE2-Fc K31W variants are more potent than wild-type hACE2-Fc, produced in CHO cells, in the described setting.

## Discussion

In the ongoing COVID-19 pandemic, there is still a need for widely-available therapeutic options. The applicability of soluble recombinant hACE2 proteins as decoys to block the binding of SARS-CoV-2 to human cells has been studied by multiple groups^13,14,18,20,28^. Using a diverse approach of combining MD simulations, in vitro competitive inhibition assays, live-virus infection assays, and artificial neural networks in conjunction with Catalophore Halos, we finally implemented a workflow that enables a fast and efficient evaluation of a particular hACE2 variant in combination with a specific SARS-CoV-2 VOC based solely on their hACE2- and spike RBD Halo. This can be done as soon as a newly emerged SARS-CoV-2 virus strain has been sequenced. In addition, our workflow allows the identification of more effective hACE2 variants in case of newly-arising SARS-CoV-2 VOCs in the future.

Our rapid approach to evaluate the hACE2-spike RBD binding affinity consists of a multi-layered strategy. First, in case of the emergence of a new VOC or another variant of interest, based on its spike RBD sequence, a homology model and then a Halo is created. This spike RBD Halo is then used as an input for an ANN prediction together with the already existing set of Halos for our hACE2 samples or one particular Halo of a specific hACE2 variant of interest. This yields results quickly and produces a ranked list of the combinations. High-affinity candidates are then fed into the MD-model workflow to validate and refine the ANN’s prediction. Homology modeling, Halo creation, and ANN prediction take less than half an hour on standard desktop hardware.

Although our workflow benefits from its promptness and cost-efficiency, one must remember that it only covers alterations in the SARS-CoV-2 spike RBD. Potential binding affinity alterations and conformational changes of the whole trimeric spike protein due to substitutions in the spike S2 region, as shown for D614G^35^, are not covered. Furthermore, the influence of glycosylation-pattern changes can not be detected since ANN-guided preselection and MD-simulation-based predictions both use deglycosylated input structures.

During our studies, we identified hACE2 K31W, hACE2 T27Y_L79T_N330Y_K31W, and hACE2 T27Y_L79T_K31W, amongst others, as hACE2 variants that showed good binding properties for a variety of SARS-CoV-2 VOCs. High binding affinity is a crucial factor when it comes to virus neutralization capacity, and these two were well correlated in a previous study^20^. The neutralizing effects of hACE2 K31W, encompassing a lower mutational load than variants with three to five mutations, have been validated in vitro. hACE2 K31W and three other hACE2 variants, fused to the Fc region of human IgG at the *C*-terminal end, were expressed in CHO cells. To prevent unintended vasodilatory effects, we eliminated hACE2’s peptidase activity. The presence of wild-type hACE2-Fc, hACE2-Fc K31W, hACE2-Fc E35D, and hACE2-Fc T92C during one hour of VeroE6 virus infection (SARS-CoV-2 wild-type or Beta variant), followed by washing and 48 h incubation with the respective variant, strongly reduced the number of viral copies in the supernatant compared to the untreated control. At low hACE2-Fc concentrations between 3.13 and 0.78 μg/mL, the nearly complete suppression of SARS-CoV-2 spreading remained unaffected for hACE2-Fc K31W but vanished for wild-type hACE2-Fc. This observation was confirmed by the immunohistochemical analysis as an independent readout.

K31W has previously been described as a beneficial mutation to increase the hACE2-spike RBD affinity^28^. The sizeable aromatic side chain of tryptophan is likely to contribute to enhanced hydrophobic and pi-stacking interactions with residues from the spike RBD. In particular, the favorable interaction between W31 and Y489 is expected to be the key enhancing factor and has been shown to overshadow the effects of the disruption of two salt bridges (K31-D35 and K31-E484)^28^.

To evaluate the mass-production potential of our hACE2 variants, we transiently expressed hACE2-Fc K31W in *N. benthamiana* plant leaves (hACE2-Fc K31W_NB) and tested their SARS-CoV-2-neutralization potential in ELISA-based competitive inhibition assays. Compared to hACE2-Fc K31W produced in CHO cells (hACE2-Fc K31W_CHO), hACE2-Fc K31W_NB was more potent in inhibiting the binding of the spike RBD to immobilized wild-type hACE2-Fc. This is possibly due to differing hACE2-Fc glycosylation patterns. Wild-type hACE2-Fc expressed in *N. benthamiana* showed less processed complex N-glycans and a partial underglycosylation at the N90 position in recent LC–ESI–MS analyses compared to ACE2-Fc produced in a human cell line^36^. It was previously reported that most of the hACE2 substitutions of N90 and T92, which together form a consensus N-glycosylation motif, would be beneficial for binding to the spike RBD^18^ since N-glycans could hinder binding through steric clashes or electrostatic effects^37^. With a half-maximal inhibitory concentration (IC_50_) of 0.313 μg/mL (hACE2-Fc K31W_NB) and 1.442 μg/mL (hACE2-Fc K31W_CHO), both hACE2 mutants had a lower IC_50_ value compared to the wild-type hACE2-Fc, expressed in CHO cells, with 1.856 μg/mL. In summary, this data demonstrates that the production of hACE2-Fc variants with correct folding in *N. benthamiana* is possible, which is in line with previous studies^36,38^. Plant-produced soluble ACE2 variants represent a promising, cost-effective therapeutic option in the treatment of patients suffering from COVID-19.

This work presents a bioinformatic approach to assessing the binding affinity of hACE2 variants, potentially used as therapeutic decoys for COVID-19 patients, to SARS-CoV-2 variants. The systematic two-pronged strategy rapidly evaluates the binding affinity of any given SARS-CoV-2 VOC in combination with a vast number of hACE2 designs at an early stage after sequencing a SARS-CoV-2 VOC. Our workflow could potentially be applied to other viral targets, such as the MERS entry receptor DPP4. However, our system is especially useful in assessing the efficacy of a given hACE2 decoy to a new VOC at an early stage, shortening timelines for hACE2-decoy adaptation and reducing the number of samples for in vitro selection.

## Materials and methods

### Construction of the MD-simulation system

#### Preparation of spike RBD-hACE2 structures

A crystal structure of the wild-type SARS-CoV-2 spike RBD bound with hACE2 was downloaded from the PDB (ID: 6M0J). Energy minimization was performed using a short steepest-descent minimization followed by simulated annealing (timestep 2 fs, atom velocities scaled down by 0.9 every 10th step). For this purpose, the AMBER14 force field^39^, applying an 8 Å force cutoff, was implemented. The minimized structure was implemented as a template for homology modeling. hACE2 mutants with experimentally determined binding affinities (Table S1) were used to validate the ESF. Their structures were built by introducing the mutation into the protein sequence and subsequent homology modeling using Yasara^40^. The same homology-model experiment was performed for the wild-type spike RBD:hACE2 amino-acid sequence (ID: 6M0J) to guarantee identical handling of input structures. The final input files contained residues 333–526 of the respective SARS-CoV-2 spike RBD and residues 19–615 of hACE2 coordinating one zinc ion.

#### System setup and training

The system was established as described previously^30^. Initial structures were solvated in a cuboid box with periodic boundaries. The cell was filled with water at a density of 0.997 g/cm^3^, ionizable groups were protonated according to pH 7.4 and 0.9% NaCl counter ions were added. Energy minimization took place before and after each simulation phase to clear bumps and adjust the covalent geometry. For this purpose, the same preparation procedure as described above was implemented. MD simulations were carried out using the AMBER14 force field with automatic parameter assignment by AutoSMILES^41^ at 310 K and 1 bar. The spike RBD:hACE2-complex structure, as well as the unbound hACE2 structure, were simulated for 200 ps. Energy snapshots were extracted every 200 fs during the simulation.

### hACE2 screening

#### hACE2 design

hACE2 mutations were initially selected according to visual inspection with a focus on affinity optimization using a spike RBD:hACE2 crystal structure from the PDB (ID: 6M0J), and the collection was extended by the top-ten high-affinity hACE2 variants, determined by Chan et al*.* in flow-cytometry binding affinity experiments^18^ and combinations of the two approaches (Fig. S2).

#### Structure preparation

To examine binding affinities between the suggested hACE2 variants and SARS-CoV-2 variant of concern (VOC) spike RBDs, suggested hACE2 mutations and spike RBD residue changes reported by the WHO for Beta, Delta, Omicron BA.1, Omicron BA.2, Omicron BA.2.75, Omicron BA.3 and Omicron BA.4/BA.5^42^ were manually incorporated into the wild-type sequence (PDB: 6M0J), and homology models were built with Yasara. The final input files contained residues 333–526 of the respective SARS-CoV-2 spike RBD and residues 19–615 of ACE2 coordinating one zinc ion.

### MD-simulation

The model-predicted value of Δ*G* is computed from the linear combination of two energy contributions, one term coming from van der Waals (vdw) and another from electrostatic forces (elec). The energy contributions are differences of the ensemble averages of bound and unbound configurations. In the bound case, the hACE2 environment includes water molecules and spike RBD atoms, whereas, in the unbound state, hACE2 is exclusively surrounded by water molecules within the simulation box. While the energy differences come from MD ensembles, the weights for these contributions were determined using a set of 43 structures along with experimental K_D_-values with simulation parameters optimized as described previously^30^. For our model calculations, we used 200-ps simulations with 50 replicates per variant, which led to Eq. (1).

1 $$\Delta G=0.765\Delta {E}^{vdw}+0.024\Delta {E}^{elec}$$

It should be noted here that the weight for the electrostatic term is relatively small. This leads to a small contribution from the electrostatic energy differences to Δ*G* since energy differences in both terms are of the same order of magnitude. Our previous study showed that electrostatic interaction energies are too broadly distributed in each simulation run to lead to a good-enough signal-to-noise ratio for the corresponding bound-unbound difference. As a result, the model fit reduced the contribution of this term significantly in order not to destroy the correlation between predicted Δ*G* and the gauging data.

### Model validation

#### *Comparison to experimental EC*_*50*_* values*

A subset of the hACE2-variant collection was tested in in vitro binding affinity experiments (see below). The correlation between predicted Gibbs free energy values from the simulation and logarithmic EC_50_ values was evaluated by calculating *R*^2^.

### hACE2 production

#### Construction of expression plasmid

The full-length cDNA of human ACE2 (GenBank Accession No. AF291820) was purchased from Sino Biological Inc. (Beijing, China). The cDNA-encoding hACE2 (residues 19–720) for Chinese-hamster-ovary (CHO) expression was amplified by polymerase chain reaction (PCR) and cloned into the expression vector pYD11SP. The insert was fused to the human IgG1 signal peptide at the *N*-terminus and to the Fc region of human IgG1 at the *C*-terminal end. The designed mutations were introduced by PCR to generate hACE2 mutants with higher potency. To eliminate hACE2 peptidase activity, H374N and H378N mutations were introduced by overlap extension PCR.

For expression in *N. benthamiana*, coding sequences of hACE2 (K31W)-Fc were amplified using the following primers:

- Forward primer 5′-GCC GGT CTC GCT TCA GGC ATG GAT CCA TGT CCA CCA TTG AGG AAC AGG CCA AGA CA-3′
- Reverse primer: 5′-CTT TTA GCT CAG CAT TCT GCT TTT GAG CTC TCA TTT ACC CGG AGA CAG GGA GAG G-3′.

Sequences were inserted in the pSCMP plant-expression vector via *Bam*HI- and *Sac*I-restriction sites using In-Fusion® HD Cloning Plus PCR Cloning Kits (Takara Bio USA, Inc., San Jose, CA, USA), resulting in pSCMP-ACE2(K31W)-Fc fused to barley alpha-amylase 2 signal peptide.

#### Expression of soluble hACE2-Fc

CHO cells were maintained in FreeStyle™ F17 medium (Invitrogen, Waltham, MA, USA) supplemented with 4 mM glutamine and 0.1% Kolliphor P-188 (Sigma, St. Louis, MO, USA). The cells were grown at 37 °C in shake flasks on an orbital shaker set to 120 rpm in a humidified 5% CO_2_ incubator. For transfections, cells were seeded at 1.0 × 10^6^ cells/mL on day 0 and transfected with Polyethylenimine MAX linear (LPEI MAX, MW 25, Polysciences Inc.) on day 1. Briefly, 80 µg DNA plasmid and 133 µL LPEI MAX stock solution (3 mg/mL) were diluted with 5 mL FreeStyle™ F17 medium, respectively. The diluted DNA and LPEI-MAX were combined and incubated at room temperature for 3 min. 10 mL of the mixture was added to 90 mL overnight CHO cell culture. At 4 to 24 h post-transfection, 3 mL of tryptone N1 (Organotechnie, La Courneuve, France) was added. The conditioned media were collected 72 h after transfection to purify soluble hACE2-Fc proteins.

*N. benthamiana* was cultivated in a growth room under a 16 h light:8 h dark photoperiod at 22 °C and 50% relative humidity. The binary construct pSCMP-ACE2(K31W)-Fc was introduced into *Agrobacterium tumefaciens* strain AGL1 by the freeze–thaw method. The AGL1 strains were inoculated in selective liquid YEP media with 50 μg/mL rifampicin, 50 μg/mL carbenicillin, 100 μg/mL kanamycin at 28 °C in a shaking incubator at 200 rpm for two days. For transient expression, the AGL1 pellet harboring pSCMP-ACE2-Fc was resuspended and diluted in 1 × infiltration buffer containing 10 mM 2-(N-morpholino) ethanesulfonic acid (MES), 10 mM MgSO_4_, 5% glucose, 200 μM acetosyringone, at pH 5.3 to an OD_600_ of 1.0. The AGL1 strains were vacuum-infiltrated into the 6-week-old *N. benthamiana* plant leaves and maintained at 22 °C growth room. The leaf tissue was harvested 4 days post infiltration (dpi) for protein-expression extraction and purification.

#### Purification of hACE2-Fc

Cleared conditioned media from CHO cells transfected with soluble hACE2-Fc were supplemented with 0.5 mL equilibrated MabSelect™ PrismA Resin (GE Healthcare, Chicago, IL, USA) and incubated in a fridge with shaking overnight. The resin was collected on a chromatography column and washed with 50 mL of buffer A (20 mM sodium phosphate, 150 mM NaCl, pH 7.2). The proteins were eluted with buffer B (0.1 M glycine, pH 3.5). The eluate was immediately neutralized with 1 M Tris, pH 10.6. The hACE2-Fc-containing fractions were pooled, and the storage buffer was changed to 1 × PBS. The concentrations of purified hACE2-Fc proteins were determined by Bradford assay.

For purification of recombinant soluble hACE2-Fc expressed from *N. benthamiana*, 50 g of frozen leaves was extracted in ice-cold lysis buffer (1 × PBS containing 10% glycerol, 1% PVP, 0.5% TritonX-100, 1 mM PMSF, 0.01% β-me, 0.5% protease inhibitor cocktail) at a ratio of 1:2 (w/v) and mixed in a Magic bullet blender (Homeland Housewares, Los Angeles, CA, USA) by applying three cycles of 30 s at a 30 s interval. The crude extract was then incubated in a fridge with shaking for 30 min before centrifuging at 13,000×*g* for 25 min at 4 °C. The pH of the extract was adjusted to pH 7.2 followed by centrifugation at 13,000×*g* for 25 min at 4 °C. The cleared extract was used to purify soluble hACE2-Fc as described above.

### Cell culture and virus stocks

African green-monkey kidney-epithelial cells VeroE6 (Biomedica, Vienna, Austria) were cultivated in Gibco’s Minimum Essential Medium (MEM) supplemented with Earle’s Salts and L-glutamine (all from Thermo Fisher Scientific, Waltham, MA, USA) with 5% fetal bovine serum (FBS; Thermo Fisher Scientific) and 1% penicillin/streptomycin (Thermo Fisher Scientific), in the following referred to as MEM (5% FBS). Incubation at 37 °C, 5% CO_2_ if not stated otherwise.

A human 2019-nCoV Isolate (Ref-SKU: 026V-03883, Charité, Berlin, Germany) and a human SARS-CoV-2 Beta variant isolate (Ref-SKU: 014V-04058, EVAg, Marseille, France) were propagated in VeroE6 cells. TCID50 titres were determined according to the Reed Munch method^43^ and plaque-forming units (PFU) were calculated using the conversion factor 0.7, based on the ATCC-LGC standards (www.atcc.org/support/technical-support/faqs/converting-tcid-50-to-plaque-forming-units-pfu). For all infection experiments, the working stocks were diluted to a calculated multiplicity of infection (MOI) 0.0003 in MEM (2% FBS). All experimental steps with active SARS-CoV-2 virus isolates were performed under BSL-3 conditions.

### SARS-CoV-2 neutralization assay

Prior to every assay, purified hACE2-Fc solutions were freshly diluted in MEM (2% FBS) to hACE2-Fc concentrations of 0.78 µg/mL, 1.56 µg/mL, 3.13 µg/mL, 6.25 µg/mL, 12.5 µg/mL, 25 µg/mL or 25 µg/mL.

SARS-CoV-2 neutralization assays were performed similarly as described previously^44^. 24 h before the assay, VeroE6 cells were seeded (30,000 cells/well) in a 48-well plate in MEM (10% FBS). After preincubation of SARS-CoV-2 (wild-type or Beta variant) with hACE2-Fc protein (wild type or variant) in concentrations between 0.78 and 25 µg/mL for ½ h, cells were infected at a multiplicity of infection (MOI) of 0.0003 with the preincubation mix in a final volume of 200 μL per well in MEM (2% FBS). Here the dose control (DC) was sampled. After 1 h of incubation at 37 °C and 5% CO_2_, the mixture was removed and cells were washed two times with fresh medium to remove unadsorbed virus particles. Respective hACE2-Fc solutions were again added to the cells. Cells were then incubated over a period of 48 h at 37 °C and 5% CO_2_. In the assay, untreated infected cells were used as positive controls and non-infected cells served as negative controls. Remdesivir (THP Medical Products, Vienna, Austria) was applied as an additional control. In the respective wells, cells were preincubated with Remdesivir (10 µM) for 30 min prior to infection. Remdesivir was added again after the washing steps. 140 μL of the supernatant was harvested and inactivated to extract RNA and quantify viral-copy numbers via Quantitative Reverse Transcription PCR (qRT-PCR). After removal of the remaining supernatant, the 48-well plate was fixed in 4% formalin for SARS-specific immunohistochemical staining (IHC).

### RNA isolation, quantitative RT-PCR, and calculation of viral-copy numbers

The supernatant samples were inactivated by adding AVL buffer (Qiagen, Hilden, Germany), the viral RNA was isolated following the manufacturer’s protocol using the QIamp viral-RNA mini Kit (Qiagen) and RNA was eluted in 40 μL ultra-pure H_2_O. qRT-PCR of viral RNA was performed with the QuantiTect Probe RT-PCR Kit (Qiagen) using the Rotor Gene Q cycler (Qiagen). Reactions took place in a total volume of 25 μL at 50 °C for 30 min followed by 95 °C for 15 min and 45 cycles of 95 °C for 3 s and 55 °C for 30 s. The employed N1 primer set and probe, which enable the detection of N-gene of SARS-CoV-2, were recommended by the CDC at the CDC 2019-nCoV Real-Time RT-PCR Diagnostic Panel (www.cdc.gov/coronavirus/2019-ncov/lab/rt-pcr-panel-primer-probes.html).

- 2019-nCoV_N1-F 2019-nCoV_N1 Forward Primer 5′-GAC CCC AAA ATC AGC GAA AT-3′
- 2019-nCoV_N1-R 2019-nCoV_N1 Reverse Primer 5′-TCT GGT TAC TGC CAG TTG AAT CTG-3′
- 2019-nCoV_N1-P 2019-nCoV_N1 Probe 5′-FAM-ACC CCG CAT TAC GTT TGG TGG ACC-BHQ1-3′ FAM, BHQ-1.

To allow the calculation of viral-copy numbers, a commercially-available standard (ATCC VR-1986D genomic RNA from 2019 Novel Coronavirus, Lot: 70,035,624, ATCC, Manassas, VA, USA) was serially diluted and analyzed via qRT-PCR. The resulting Ct-values were plotted against ln[copy numbers] and Eq. (2), received from linear-regression analysis, was used to calculate the viral-copy numbers from the Ct-values of the samples for Primer and Probe N1. The calculated viral-copy numbers refer to a volume of 140 µL supernatant harvested after the neutralization assay.

2 $$y= -1.442 x + 35.079$$

### Immunohistochemical analysis

After removal of the supernatant and fixation of the cells with 4% formalin for 1 h at room temperature, cells were washed twice with PBS and incubated with PBS at room temperature for at least 10 min. Cells were treated with Triton X-100 (0.1% in PBS, Merck Millipore, Darmstadt, Germany) for 10 min. Cells were washed three times with PBS for 3 min. Endogene peroxidases were blocked by applying H_2_O_2_ (3% in MetOH, Merck) for 30 min. Cells were washed three times with PBS for 3 min. Samples were incubated for 1 h at room temperature with a 1:1000 dilution of primary antibody (SARS-CoV-2 (2019-nCoV) Nucleocapsid Antibody, Rabbit Mab, Cat: 40,143-R019, Sino Biological Inc.) in antibody diluent (REAL Antibody diluent, Dako Cat: S202230_2, Agilent Technologies, Santa Clara, CA, USA). Cells were washed three times with PBS for 3 min. Cells were incubated for 30 min with the secondary peroxidase-conjugated anti-Rabbit antibody using the REAL EnVDetectSys Perox/DAB +, Rb/M (Agilent Technologies) as a detection system. Cells were washed three times with PBS for 3 min. The cells were incubated with 100 µL Substrate-Chromogen (EC substrate-Chromogen, Dako, Cat: K346430–2, Agilent Technologies) until optimal staining of viral infected cells was reached, but not longer than 3 min. The reaction was stopped by washing with PBS. High-quality images were obtained using a light microscope (40 × magnification) in combination with the Jenoptik Gryphax Avior microscope camera and the Jenoptik Gryphax software (both from Jenoptik, Jena, Germany).

### Binding affinity assay

The wells of microtiter plates were coated with 100 μL of 2 μg/mL recombinant His-tagged SARS-CoV-2 spike RBD protein in carbonate buffer, pH 9.6, overnight at 4 °C. The next day, the coating solution was removed and the plate was washed three times with washing solution PBST (PBS + 0.05% v/v Tween20). The plate was blocked using 300 μL of 5% skim milk in PBST solution for 1 h at 37 °C. The blocking solution was completely discarded and the plate was washed three times with the washing solution. Soluble hACE2-Fc proteins were serially diluted with PBST solution containing 0.1% BSA. 100 μL of hACE2-Fc with each concentration was added into the wells and incubated at 37 °C for 1 h. The plate was washed three times with the washing solution. 100 μL of HRP-conjugated anti-Fc antibody solution (1:10,000) was pipetted to each well and incubated for 40 min at 37 °C. The antibody solution was removed and the plate was washed three times with washing solution. 100 μL of TMB substrate (Biopanda Diagnostics, Belfast, United Kingdom) was added to each well and incubated at room temperature for 5–10 min. After sufficient color development, 50 μL of stop solution (2 N H_2_SO_4_) was added to the wells. The absorbance (optical density, OD) was read at 450 nm. Binding affinities were analyzed using GraphPad Prism.

### ELISA-based competitive inhibition assay

The wells of microtiter plates were coated with 100 μL of 2 μg/mL recombinant wild-type hACE2-Fc protein in PBS buffer overnight at 4 °C. The next day, the coating solution was removed and the plate was washed three times with washing solution PBST. The plate was blocked using 300 μL of 5% skim milk in PBST solution for 1 h at 37 °C. HRP-conjugated recombinant SARS-CoV-2 spike RBD protein was prepared at a concentration of 200 ng/mL in PBST with 0.1% BSA. The blocking solution was completely discarded and the plate was washed three times with the washing solution. Soluble hACE2-Fc proteins were serially diluted with PBST solution containing 0.1% BSA and mixed with HRP-RBD at a ratio of 1:1. 100 μL of each mixture was added to the wells and incubated at 37 °C for 30 min. The plate was washed four times with the washing solution. 100 μL of TMB substrate was added to each well and incubated at room temperature for 5–10 min. After sufficient color development, 50 μL of the stop solution (2 N H_2_SO_4_) was added to the wells. The absorbance (optical density, OD) was read at 450 nm. Binding activities were analyzed using GraphPad Prism.

### Generation of ANN training data

#### Sequence data preparation

Sequences used for the preparation of ANN training data included spike RBD and hACE2 sequences either retrieved from visual inspection, literature research^18,45^, or spike protein sequences that were available at GISAID^46^ by January 4th, 2022. In addition to 39 specific spike RBD sequences obtained from a list of 145 representative spike protein sequences available at GISAID, spike RBD sequences (residues 319–541) were extracted from 1.3 million non-redundant spike protein sequences (out of 6.7 million entries) by pairwise alignment to the wild-type spike RBD and analyzed employing in-house tools in Python. For the ANN training data set, 1077 specific spike RBD sequences without any insertions or deletions and containing at least two mutations in reference to the wild-type spike RBD were considered. Due to the large number of specific spike RBD sequences bearing two mutations, these were additionally restricted to sequences occurring at least twice in the list of 6.7 million entries. Including spike RBD mutations derived from literature research^45^ and current VOCs, a total of 1165 spike RBD sequences were used for the training of the ANN. hACE2 mutations were retrieved either from visual inspection, literature research^18^, or a combination of both, resulting in 95 distinctive sequences. A list of the spike RBD-hACE2 pairs used for training the ANN is provided in Table S2.

Homology models of the respective spike RBD-hACE2 complexes were created and binding affinities for spike RBD (residues 333–526) and hACE2 (residues 19–615) were predicted via MD simulations as described above (“ACE2 screening”). Additionally, the homology models were used to calculate spike RBD Halos and ACE2 Halos.

#### Procreation of catalophore hACE2 halos and spike RBD halos

As described previously^30,47^, a Catalophore Halo is a multivariate property field composed of a collection of points in Cartesian space discretized onto an equidistant grid annotated with currently 19 physicochemical and statistical properties (e.g. electrostatics, hydrophobicity, flexibility, potential energies, hydrogen-bonding potential, or dissolvability) that are projected by a biomolecule into its surroundings.

Spike RBD-hACE2 homology models were deposited in the CATALObase platform^47^ and used as input data for calculating hACE2- and spike RBD Halos. This was achieved by a Yasara^48^ structure-preparation step combined with a Halo-creation and -annotation step. The latter was performed using a modified version of the AutoGrid tool that is part of the Autodock^49^ suite, version 4.2.3. The 3D point clouds generated with a grid spacing of 0.75 Å cover the entire outer molecular surface of either spike RBD (for spike RBD Halos) or hACE2 (for hACE2-Halos) with a thickness of 5 Å. Molecular surfaces were hereby defined by a probe radius of 1.4 Å around the atoms’ vdw radii. Focusing on the binding interface region, Halos were further restricted to a maximum distance of 5 Å to the atoms of the respective binding partner with corresponding vdw radii and were cropped down by the space which is occupied by these atoms plus their vdw radii. Consequently, the binding partner only influences the shape but none of the 19 physicochemical and statistical properties of the Halo given by the underlying biomolecule. Ligand-atom types and properties used for the annotation of point clouds were: carbon, H-bond donor hydrogen, non-H-bonding nitrogen, H-bond acceptor oxygen, H-bond acceptor sulfur, desolvation potential, electrostatic potential, aromatic carbon, phosphor, accessibility, hydrophobicity, flexibility, positioning of chains, sulfur, bromine, chlorine, fluor, iodine.

### Artificial neural network

#### Idea and intention

We trained an ANN on our MD-simulation data, augmented by experimental data for the spike RBD-hACE2 binding affinity where appropriate. The ANN uses the Catalophore Halos of both the spike RBD and the hACE2 for predicting the binding affinity based on Halo information alone (i.e., without direct reference to sequence or structure). Since getting model results this way is many orders of magnitude faster than running an MD-simulation for the spike RBD-hACE2 interaction, we can employ the ANN model to get an extremely efficient estimate for many variants of both spike RBD and hACE2. The cost of inference for a single variant of hACE2 essentially amounts to the computation of the Halo, which is a short addon to preparing the mutated hACE2 structure that serves as an input for the binding affinity MD simulation.

Based on numerous ANN-predictions, we can test many more possible variants of hACE2, compile a ranked list of the results, and then feed the most promising candidates into the MD-pipeline for validation. This serves two purposes: first of all, we validate the ANN model even further, and second of all, it provides us with predictions that have a reliability validated by MD-model gauging runs via the ESF. Overall, this approach allows us to choose a much more interesting and potentially representative sampling pattern for our hACE2-design approach than an unguided set of runs of our MD-simulation setup would provide.

#### Initial pre-omicron spike RBD experiment

Using a dataset of 1,049 spike RBD-hACE2 pairs with spike RBD amino acid exchange counts ranging from one to 20 exchanges (for a more detailed breakdown see Table S3 in the supporting information) compared to the wild-type spike RBD and a single Omicron BA.1 example, the model was trained using a random subset of 120 samples for validation. With the parameters from the epoch with the lowest validation loss for inference, the model was tested on a previously unseen set of 300 spike RBD-hACE2 combinations. Here it reached a mean error of 1.05 kJ/mol, with a maximum error of 4 kJ/mol. The Pearson correlation for this set was 0.69.

#### Initial pre-omicron hACE2 experiment

A subset of 50 unique hACE2 variants was isolated and removed from the set. The remaining set was again split into training and validation. Using the parameters that produce the lowest validation loss during training for inference, the influence of these hACE2 variants was predicted. The average prediction error was 1.2 kJ/mol, and the maximum error was 4.8 kJ/mol.

The chosen subset of hACE2 amino-acid exchanges induces a mean variance of 3.72 kJ/mol on calculated spike RBD-hACE2 binding affinities, compared to the spike RBD binding with the wild-type hACE2 alone. From this, we conclude that the model explains a large share of the variance induced by the hACE2 amino acid exchanges. Figure S5 shows the prediction error as a function of the ground-truth energies of the predicted samples.

Fig. S6 shows the distribution of samples in the entire training set with respect to their binding affinity values. The vast majority of samples is concentrated around − 60 to − 50 kJ/mol, with a strongly underrepresented minority found at binding affinities lower than − 65 kJ/mol. Since a high accuracy in the low-energy regime is desirable and most important, the machine-learning-specific challenge lies in avoiding a network bias towards the mean of the distribution and instead transferring information learned from the bulk of the distribution to the low-energy outliers. The Omicron BA.2 experiment described above shows better performance than copying values from or regressing to the mean of the closest samples seen during training for high-energy values, thus indicating success in predicting the strongly underrepresented labels.

#### Network architecture

The ANN used for predicting binding affinities is a 3D convolutional neural network (CNN) which we named “Tandem ZipperNet”. The network takes two voxelized Halos as inputs and outputs predictions of Gibbs free-energy values (Δ*G*). The overall architecture consists of three blocks; the first block uses separated convolutions, processing the input Halos of spike RBD and hACE2 binding sites individually but with shared weights. This layer is supposed to increase spatial independence on the data and reduce the imbalance resulting from different numbers of unique spike RBD and hACE2 variants in the training data.

These separated convolutions are then followed by a single convolution acting on the output volume stacked along the channel dimension, effectively joining the Halos spatially while keeping the channel size, thus functioning as the “Zipper”. A third convolutional block acts on the joined data, first increasing channels and later introducing a bottleneck at the channel dimension. The output is subsequently flattened and fed into a multi-layer-perceptron (MLP). For more information see “Network Input Data” and “Network Regularization and Augmentation” in the supporting information.

### Statistics and display

Statistical analyses were performed and plots were generated using GraphPad Prism 9 as well as Seaborn and Matplotlib. All p-Values were computed using scipy.stats.pearsonr, where the p-Value is computed using Student’s exact distribution of r as noted in the corresponding documentation. Coordinate files for Fig. 3 were generated in PyMOL. Images were rendered using Blender and Open3D.

## Supplementary Information

Supplementary Information 1.

Supplementary Information 2.

Supplementary Information 3.

## Data Availability

Publicly available datasets were analyzed in this study. This data can be found here: https://www.gisaid.org/. Input and final structure files as well as Pandas Dataframes of interaction energies exported as Python Pickle files generated within this work are available for download at https://doi.org/10.6084/m9.figshare.19904953.
