# Supplementary material for: Optimizing variant-specific therapeutic SARS-CoV-2 decoys using deep-learning-guided molecular dynamics simulations
Source: Sci Rep. 2023 Jan 14;13:774. doi: 10.1038/s41598-023-27636-x (PMC9840421; doi:10.1038/s41598-023-27636-x)
Supplement: Supplementary file 3 — Supplementary Information 3. [file 41598_2023_27636_MOESM3_ESM.pdf]

Supporting information

Optimizing variant-specific therapeutic SARS-CoV-2 decoys using deep-learning-guided molecular dynamics simulations

Katharina Köchl[1,2], Tobias Schopper[1], Vedat Durmaz[1], Lena Parigger[1], Amit Singh[1,3], Andreas Krassnigg[1], Marco Cespugli[1], Wei Wu[4], Xiaoli Yang[4], Yanchong Zhang[4], Welson Wen-Shang Wang[4], Crystal Selluski[4], Tiehan Zhao[4], Xin Zhang[4], Caihong Bai[4], Leon Lin[4], Yuxiang Hu[4], Zhiwei Xie[4], Zaihui Zhang[4], Jun Yan[4], Kurt Zatloukal[5], Karl Gruber[1,3,6], Georg Steinkellner[1,3*] & Christian C. Gruber[1,2,3,6*]

[1]Innophore GmbH, 8010, Graz, Austria

[2]Austrian Centre of Industrial Biotechnology, 8010, Graz, Austria

[3]Institute of Molecular Bioscience, University of Graz, 8010, Graz, Austria

[4]SignalChem Lifesciences Corp., 110-13120 Vanier Place, Richmond, BC V6V 2J2, Canada

[5]Diagnostic- and Research Center for Molecular Biomedicine, Institute of Pathology, Medical University of Graz, 8010, Graz, Austria

[6]Field of Excellence BioHealth - University of Graz, 8010, Graz, Austria

*Correspondence should be addressed to (christian.gruber@innophore.com or georg.steinkellner@innophore.com)

Table of contents

MD-simulation **2**

Artificial Neural Network **5**

Motivation. 5

Network Input Data. 5

Training the ANN for hACE2 candidate pre-selection. 7

Results. 7

Network Regularization and Augmentation. 10

SARS-CoV-2 neutralization assay **12**

MD-simulation

Table S1 | Binding activities of wild-type hACE2-Fc and hACE2-Fc variants with immobilized recombinant SARS-CoV-2 spike protein RBD in the ELISA binding affinity assay and predicted Gibbs free binding energies (ΔG_{pred}) from the MD simulations. Binding half maximal effective concentration (EC_{50}) values were calculated using GraphPad prism. ΔG_{pred} values were predicted using the empirical scoring function (ESF).

hACE2 mutation	EC_{50} (nM)	ΔG_{pred} (kJ/mol)
WT	0.89	-51.22
K31W	0.16	-58.01
N330Y	0.24	-54.08
T27Y, L79T, E35D	0.14	-59.43
T27Y, L79T, K31W	0.11	-60.32
T27Y, L79T, N330Y, E35D	0.18	-57.41
T27Y, L79T, N330Y, K31W	0.12	-55.91
T27Y, L79T, N330Y, N90E	0.08	-58.77
T27Y, L79T, N330Y, T92C	0.12	-59.61
T27Y, L79T, N330Y, T92C, E35D	0.18	-58.12
T27Y, L79T, N330Y, T92S	0.14	-61.06
T27Y, L79T, N90E	0.17	-57.35
T27Y, L79T, T92C	0.15	-57.20
T27Y, L79T, T92C, E35D	0.11	-57.53
T27Y, L79T, T92S	0.22	-55.72
T27Y, N330Y, A386L	0.20	-62.22
T92C, K31W	0.30	-57.12
L79R, Q24E	2.04	-51.66
N330R	2.39	-52.62
R393E	0.78	-56.18
L79Y	0.62	-54.67
T27Y, L79T, N330Y, A386L	0.24	-61.50

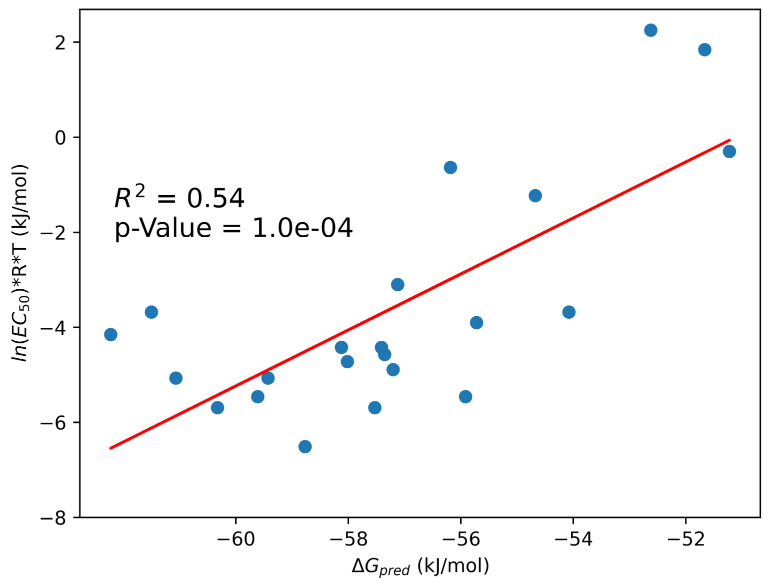

Fig. S1 | Validation of the empirical scoring function (ESF) results using an experimental half maximal effective concentration (EC_{50}) test set. To calculate the correlation between Gibbs free binding energies (ΔG_{pred}) and EC_{50} values, logarithmic EC_{50} values were adjusted under consideration of temperature (T) and the gas constant R.

Table S2 | List of the spike RBD-hACE2 pairs used for training the ANN is provided in Table S2. The full list of RBD-hACE2 pairs is provided as a machine readable CSV (Comma-separated values) file Supplementary_Table_S2.csv.

RBD Mutations	hACE2 Mutations	RBD Source	hACE2 Source	Pre-Omicron RBD Experiment	Pre-Omicron hACE2 Experiment	Omicron BA.2 Experiment
K417N, E484K, N501Y	H34A	Beta Variant, WHO	Chan et al. (2020)	X	X	X
K417N, E484K, N501Y	L79I	Beta Variant, WHO	Chan et al. (2020)	X	X	X
. . .						

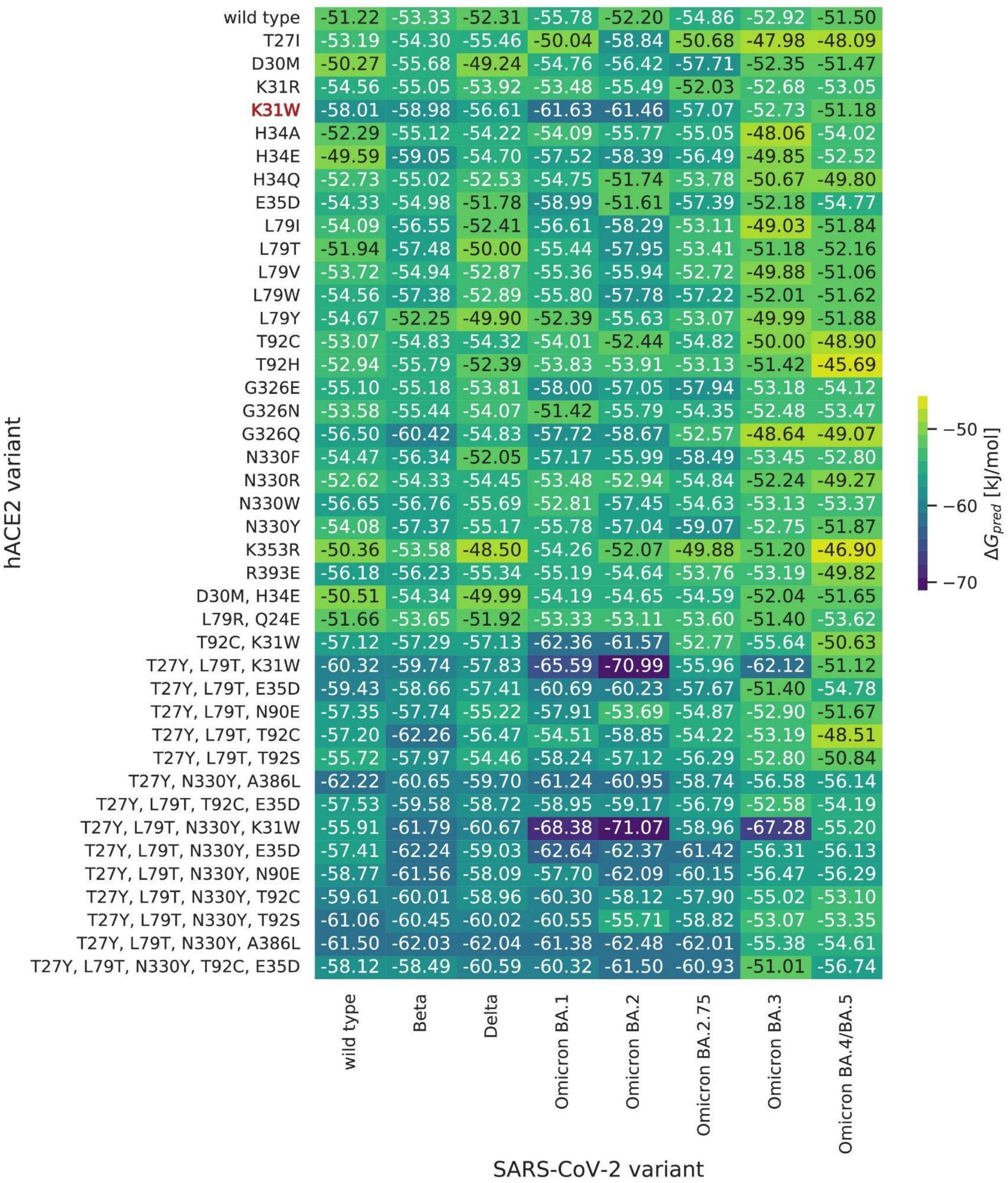

Fig. S2 | Preselection of hACE2 variants with Gibbs free energy predictions. hACE2 variants selected either by visual inspection, literature research or a combination of both with the goal to enhance the binding affinity between SARS-CoV-2 RBD and hACE2. Van der Waals (vdw) and electrostatic (elec) energies from the MD simulation in combination with optimal fitted weights ($\omega^{elec} = 0.024$, $\omega^{vdw} = 0.765$) from the training set resulted in predicted Gibbs free energies (ΔG_{pred}).

Artificial Neural Network

Motivation.

We employed the ANN to predict new viable hACE2 candidates. Since randomly searching through the entire mutation landscape using molecular-dynamics simulations is computationally infeasible, the binding affinities of all possible single-point mutants of the wildtype-hACE2 were predicted by the ANN, of which the most promising candidates were then analyzed via the empirical scoring function.

Network Input Data.

The input channels were decided on to be the following set of Halo properties:

- "e" representing the electric field
- "A" : representing the force-field experienced by aromatics
- "HP" : The hydrophobicity-field
- "HD" : Hydrogen-bond donor field
- "d" : Desolvation field
- "I" : Iodine field
- "BF": Flexibility field
- "NA": Nitrogen as H-bond acceptor field

Halos only depend on surface geometry and are independent of the exact underlying structure of their respective proteins, which allows the ANN to be agnostic to the protein-structure. Fig. S3 shows an example set of Halos. To see their relation to the protein structure, we refer to Fig. 3.

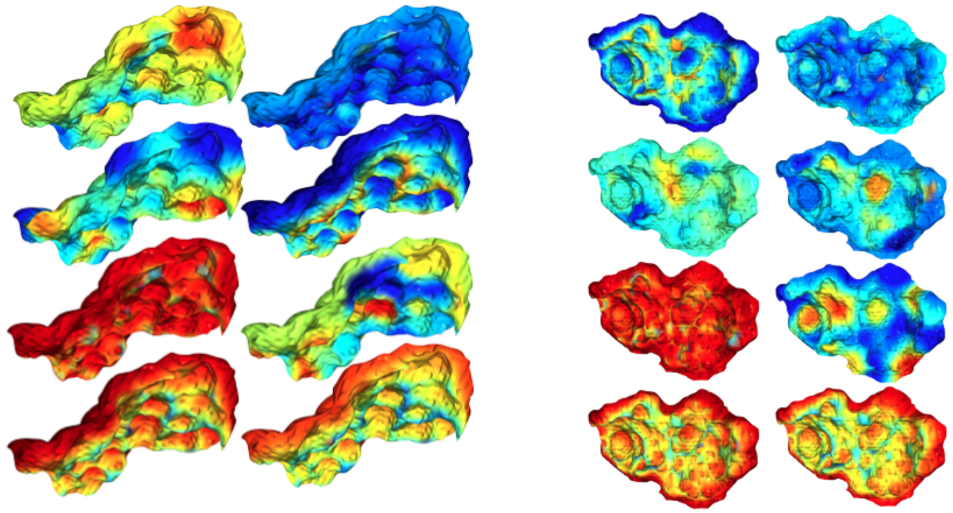

Fig. S3 | Example Halos of the eight input-channels for hACE2 (left) and RBD (right) rendered in Open3D. First column, from top to bottom: electrostatics (e), hydrophobicity (HP), hydrogen-bond donor (HD), nitrogen as hydrogen-bond acceptor (NA). Second column, from top to bottom: Iodine (I), desolvation field (d), flexibility/uncertainty field (BF), aromaticity (A).

This decision was made by correlation studies independent from the present work, deciding on this set of eight largely uncorrelated features. Preliminary ablation studies have shown that, if each property is used separately one by one, the hydrophobicity field has the strongest predictive power, outperforming all others by 0.1 to 0.15 kJ/mol validation error. On the other hand, all of the eight channels alone are sufficient to produce meaningful predictions, with the *"nitrogen as H-bond acceptor"* field, which for hACE2 and RBD Halos seems to correlate mainly with their depth-profile perpendicular to the attachment plane, exhibiting unstable and slow training as well as worse convergence on validation metrics, suggesting that the surface geometry alone is important but not sufficient. The best performing combination of fields evaluated for the prediction of new hACE2 variants has been determined to be, in descending order of importance:

1. Hydrophobicity field
2. Hydrogen-bond donor field

3. Aromatic field
4. Electric field

The voxelized Halos were padded to a size of 36x38x33 voxels, at a grid spacing of 1.4 Å between neighboring voxels. A visualization of the Halos for the properties listed above is shown in Fig. S3.

Training the ANN for hACE2 candidate pre-selection.

The validation set for the experiments was chosen to be any combination containing hACE2 variants with the amino-acid exchanges T27Y and L79T. This results in a set of 70 combinations, and includes the three best-performing samples, which contain the two highest-affinity hACE2 variants “T27Y, L79T, N330Y, K31W” and “T27Y, L79T, K31W”, leaving the lowest ΔG values in the training set at -67.1 kJ/mol. Using this validation set to learn how the ANN behaves when shown out-of-distribution values of ΔG, the model training was stopped when the validation loss was minimal, under the requirement that all high-performers were assigned ΔG values lower than -60 kJ/mol.

This approach was chosen since this way the network was trained so that it would have been able to predict the current highest-affinity hACE2 mutation without having seen them during training.

Results.

Out of all single-point hACE2 mutants, the ANN only found five values with ΔG values lower than -60 kJ/mol. For ΔG values lower than -59 kJ/mol it found nine, for ΔG values lower than -58 kJ/mol, 18 candidates were found. The candidates with ΔG values < -60 kJ/mol successfully

gave three new hACE2 mutations with values < -63 kJ/mol, one of which (K31E) even reached state-of-the art performance of -71 kJ/mol. Note that the previous highest-affinity hACE2 mutant was built using four amino-acid exchanges, versus just a single one in this case. Fig. S4 shows the distribution of the obtained MD results in dependence of the ANN prediction, and shows a clear trend towards higher MD affinities for higher predictions.

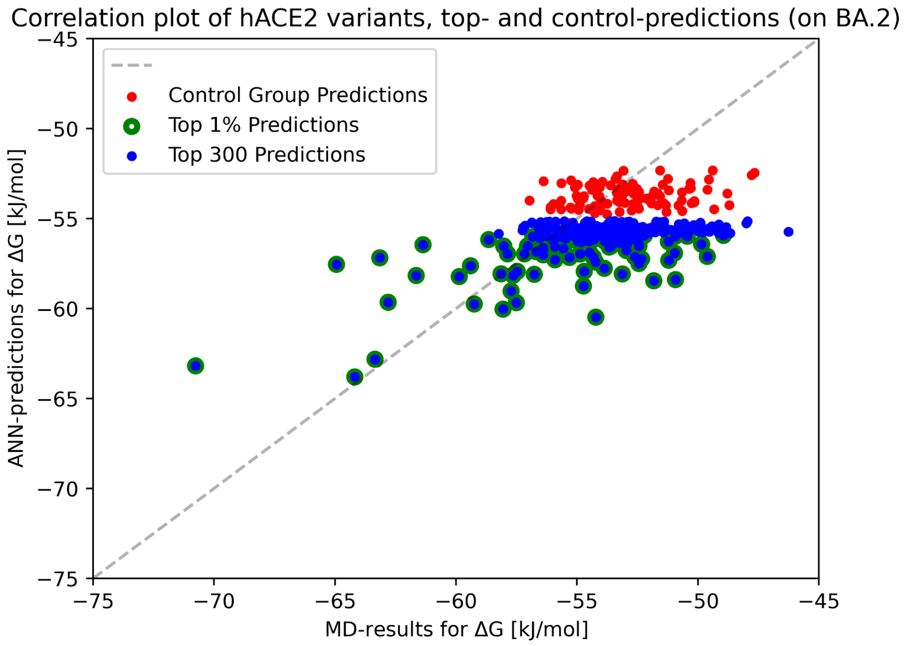

Fig. S4 | Correlation-plot between ANN predictions and MD-results for single-point hACE2 mutants. The control group shows 100 randomly sampled single-point amino-acid exchanges from all possible single-point mutations. Blue points indicate the 300 highest affinities predicted by the ANN from all single-point variants, green outlines indicate that points belong to the top 1% (110) of ANN predictions.

Table S3 | Detailed composition of the initial artificial neural network training-set.

RBD Mutations	Count	ACE2 Mutations	Count
0-5 Mutations	621	1 Mutation	120
6-10 Mutations	187	2 Mutations	15
10-15 Mutations	176	3 Mutations	30
15 - 20 Mutations	64	4 Mutations	35
28 Mutations	1	5 Mutations	5
—-	—-	Wild type	844

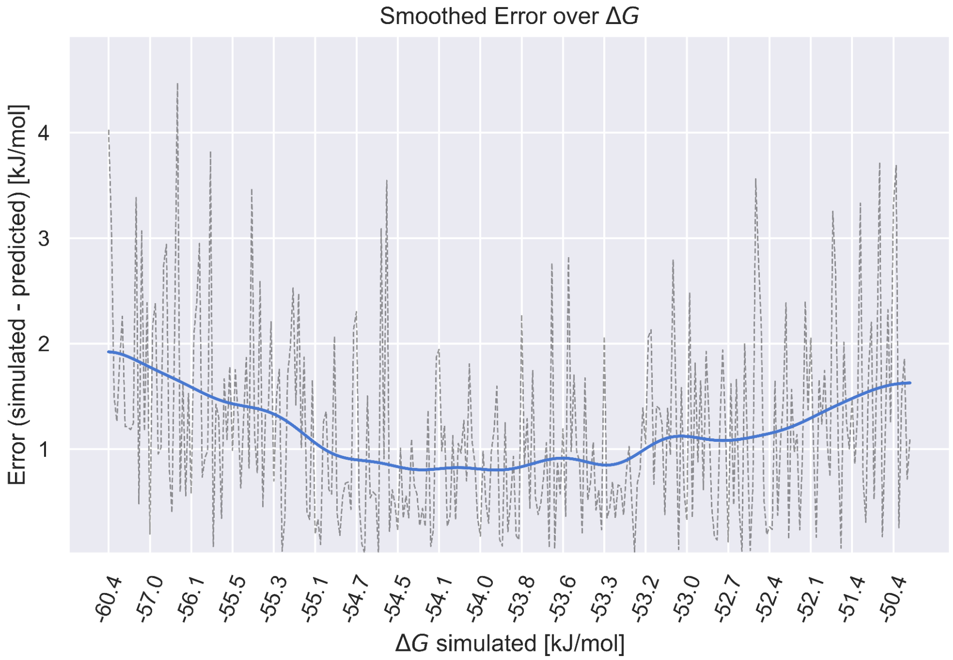

Fig. S5 | Qualitative representation of model error dependence on Gibbs free-energy values (ΔG) from the MD simulations.

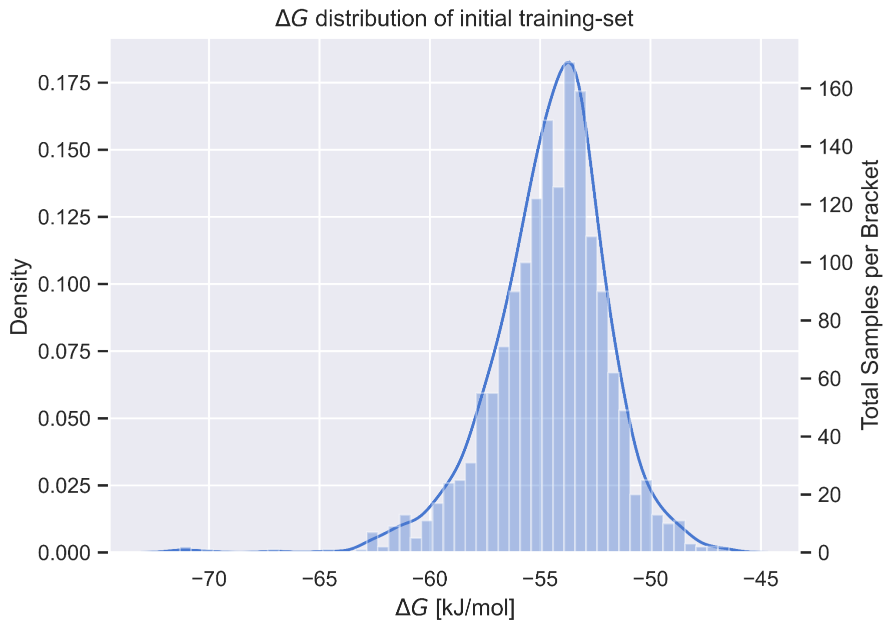

Fig. S6 | Gibbs free-energy value (ΔG) distribution of the entire training set. Strong binders with energies <-65 kJ/mol and weak binders with energies >-45 kJ/mol are severely underrepresented, yet the model still produces accurate predictions.

Network Regularization and Augmentation.

No explicit techniques for regularization were employed, only the implicit mechanisms induced by separated convolutions on the input Halos, as well as a channel-wise bottleneck in the last convolutional layer.

The data labels were augmented with an additive Gaussian-noise term with mean zero and variance 0.6 kJ/mol, which amounts to half of the uncertainty of the MD simulation itself. Furthermore, the MSE loss was again squared, leading to a stronger emphasis on rare outlier samples. This strategy was chosen over under- or oversampling to avoid memorizing outliers, as well as to keep retraining the network with the bulk of noisy samples, which adds an additional stochastic element to the loss landscape, avoiding the overfitting of common samples. Loss

values under 0.5 kJ/mol were set to zero, as these deviations were not considered physically meaningful and did not need to be emphasized further during training.

SARS-CoV-2 neutralization assay

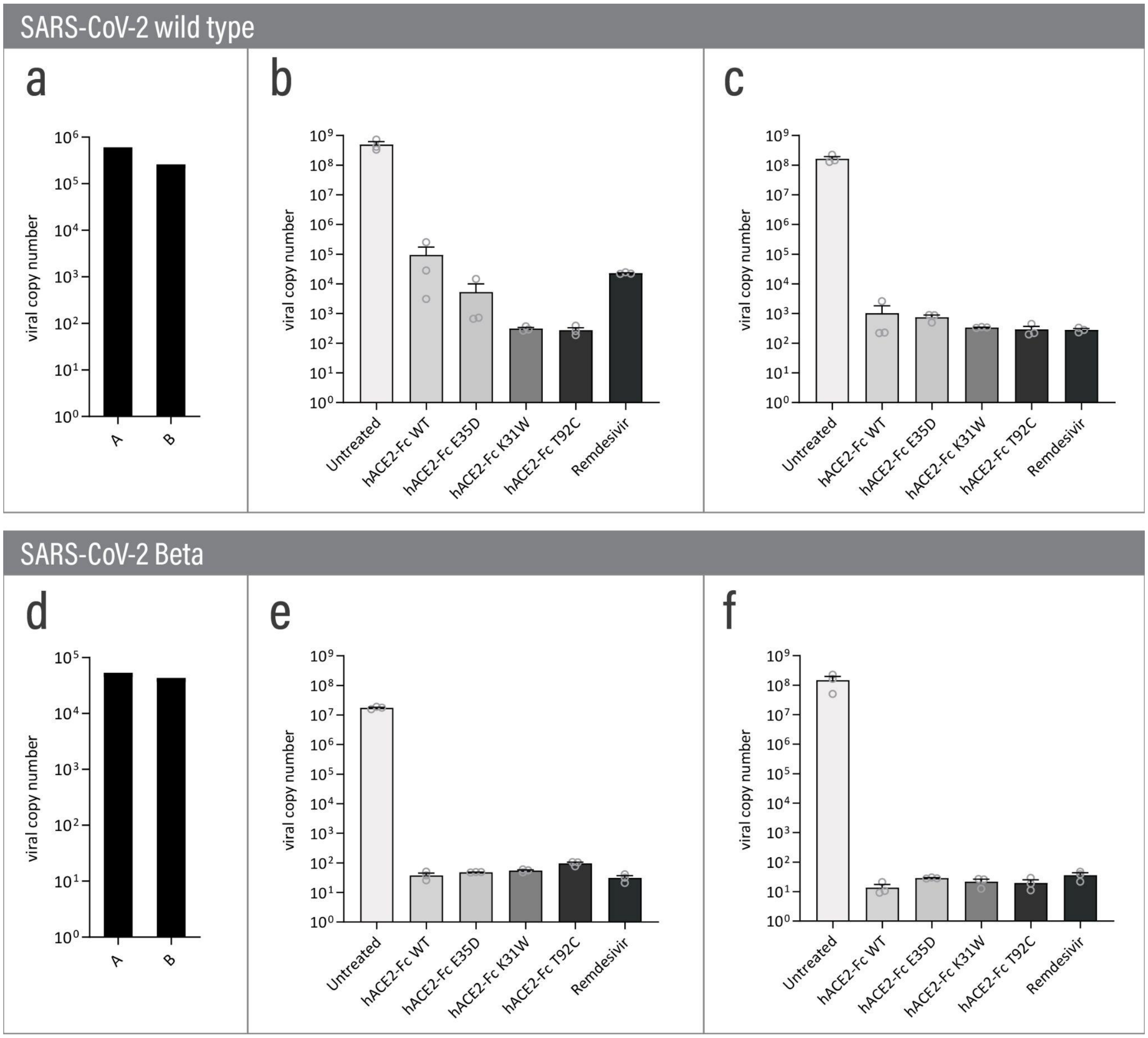

Fig. S7 | Inhibiting effect of hACE2-Fc wild type (hACE2-Fc WT) and three hACE2-Fc variants on SARS-CoV-2 (wild-type and Beta variant) infection. Each hACE2-Fc variant (25 µg/mL) was preincubated with SARS-CoV-2 (MOI: 0.0003) and then added to VeroE6 cells. Remdesivir (10 µM) treatment and infections without treatment ("Untreated") served as controls. Supernatants (SN) were collected 48 h post-infection and RNA levels were analyzed by qRT-PCR. **a**, SARS-CoV-2 wild-type virus inputs for two independent experimental series, which led to the viral titers shown in **b** and **c**. SARS-CoV-2 Beta virus inputs for two

independent experimental series are shown in **d**. The associated SN contained viral titres illustrated in **e** and **f**. qRT-PCR data are mean ± SEM of three replicates per tested condition.
